# Melittin inhibits proliferation, migration, and invasion in osteosarcoma cell lines using 2D and 3D models

**DOI:** 10.1590/1678-9199-JVATITD-2024-0053

**Published:** 2025-04-14

**Authors:** Giovana Pedro, Felipe César da Silva Brasileiro, Rui Seabra Ferreira, Aline Márcia Marques Bráz, Renée Laufer-Amorim

**Affiliations:** ¹ School of Veterinary Medicine and Animal Science, São Paulo State University (UNESP), Botucatu, SP, Brazil.; ² Center for the Study of Venoms and Venomous Animals (CEVAP), São Paulo State University (UNESP), Botucatu, SP, Brazil.; ³ Flow Cytometry Laboratory, Applied Biotechnology Laboratory, Clinical Hospital of Botucatu Medical School, Botucatu, SP, Brazil.

**Keywords:** Comparative oncology, Natural compounds, Translational medicine, Bee venom

## Abstract

**Background:**

Osteosarcoma is the most common primary bone tumor in humans. It is a locally aggressive tumor at the primary site, with metastasis being the main cause of death in patients. Studies on dogs have gained prominence in oncology, as they are valuable spontaneous models of osteosarcoma. In the context of natural compounds, biotoxins are attracting increasing research interest as new therapeutic agents against cancer, such as melittin, that represents 40 to 50% of the dry weight of bee venom, and studies have already shown its antitumor effects.

**Methods:**

We analyzed the anti-migratory and anti-invasive potential of melittin, with the wound healing and Transwell tests, apoptosis with Annexin V/IP and cell viability with the MTT test in 2D and 3D models.

**Results:**

Melittin had a cytotoxic effect on osteosarcoma cell lines, with an IC50 between 1.5 and 2.5 µg/mL. In the wound healing test and Transwell test, melittin prevented cell migration and invasion, resulting in cell death due to iodide propidium marking in canine, murine and human cell lines. Melittin exhibited cytotoxicity in a 3D model of osteospheres, with a significantly higher IC50 in this type of culture, with values between 3.5 and 4.0 µg/mL.

**Conclusion:**

We conclude that melittin has antitumor and antimetastatic properties in canine, murine and human osteosarcoma cell lines. Consequently, we believe that further research on this promising compound will facilitate its application in the development of therapeutic agents for osteosarcoma, ultimately contributing to improved survival outcomes for cancer patients.

## Visual Abstract

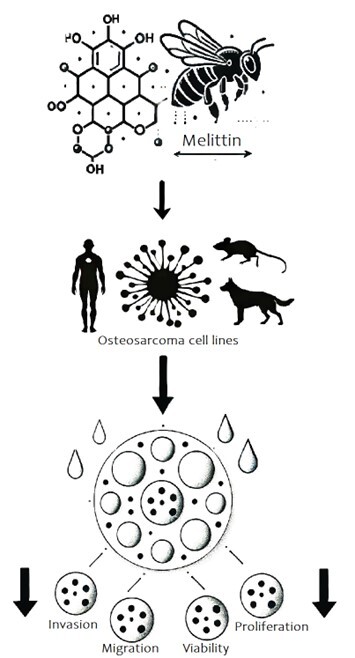

## Background

Osteosarcoma (OSA) is the most common primary bone tumor in humans. It occurs mostly in adolescents and is correlated with the peak of skeletal growth, presenting a greater risk for taller children [1, 2]. It develops mainly in the appendicular skeleton, in the metaphyseal region of long bones, and close to the growth plates. The cause of osteosarcoma is multifactorial but preexisting skeletal abnormalities and hereditary genetic predispositions are known risk factors [1, 2, 3, 4]. Genetic mutations, such as the loss of *TP53* and *RB1*, which control DNA repair and cell cycle progression, have also been identified as participants in the development of OSA [4].

OSA is a locally aggressive tumor at its primary site that can cause bone proliferation and lysis, besides metastasizing. It occurs mainly in the lung and can also affect lymph nodes, soft tissues, other bones, and other visceral organs [5]. Approximately 15 to 20% of patients who present with metastasis at the time of diagnosis die. Even with surgical treatment associated with chemotherapy, there has been no improvement in survival time in the last 15 years, with 30 to 40% of children dying from the disease [5]. In dogs, about 70 to 80% of patients with pulmonary metastasis die, with a survival time of less than 5 months, even with the treatment of choice [6, 7].

 For this reason, several animal models have been used to study the behavior and biology of OSA, such as murine models, which are extremely important for studying the metastatic cascade, in addition to the effects of antitumor agents, both in primary sites and in metastases. However, there are significant differences in spontaneous occurrence, primary tumor site, homogeneity, and growth rate [1, 8, 9]. Studies on canine OSA have gained prominence in oncology, as they are valuable spontaneous models for different tumors, since they share the same biological behavior, genetic changes, and histopathology, in addition to risk factors such as environmental exposure, genetic mutations and familial predisposition [8, 9, 10].

As dogs are a heterogeneous group of patients, they more accurately represent the population of nonconsanguineous humans than rodents do, in addition to the spontaneous primary sites being in long bones in both the femur in humans and the radius in dogs [10, 11]. Like humans, age plays an important role, large and giant breeds are those most affected by canine OSA [10]. Comparative oncology studies are needed to evaluate the effectiveness and viability of new treatments, such as natural compounds [8, 12].

Natural compounds, especially biotoxins, are attracting increasing research interest as new therapeutic agents against cancer. Biotoxins are produced by living organisms to defend against attack by predators, as is the case for bee venom, which is a complex mixture of biologically active peptides, including melittin [13].

Melittin represents 40 to 50% of the dry weight of bee venom, and is composed of 26 amino acids, water soluble and cationic [13, 14]. Studies have shown its antibacterial, antifungal, antiparasitic, and antitumoral effects. It inhibits the proliferation of tumor cells, inducing apoptosis through several mechanisms, such as changing membrane permeability, increasing intracellular Ca^2+^ [14], and acts on matrix metalloproteinases, preventing migration and metastasis. Its antitumoral mechanisms are diverse, including modulate energy metabolism, inhibit cell invasion, and increase tumor sensitivity to radio- and chemotherapy in different types of human tumor cell lines, including osteosarcoma cells [14, 15].

To better evaluate tumor properties *in vitro*, 3D models were created. The concept of 3D spheres is based on the creation of spheroid structures in which cells form several layers. This structure mimics the physical and biochemical characteristics of a solid tumor mass. This feature of 3D models is the result of appropriate cell‒cell and cell‒environment interactions, which are created to imitate the tissue structure. Another important attribute of 3D culture is its similarity to cells growing *in vivo* in terms of cell topology, gene expression, signaling, and metabolism [16].

The aim of this study was to test the antitumoral potential of melittin, in addition to its ability to inhibit cell proliferation, migration, and invasion, and to test its cytotoxic effect on osteospheres, in canine, murine and human cell lines. To our knowledge, this is the first report of an *in vitro* canine model in which melittin was used as an antitumor agent, which is currently one of the most important comparative and translational oncology models.

## Methods

### Materials and chemicals

The following chemicals and reagents were purchased from their respective manufacturers and used in this study: antibiotic antimycotic solution, gentamicin, and trypsin-EDTA solution from Gibco™ (Thermo Fisher Scientific); APC conjugated with Annexin V, Hoechst, propidium iodide, and 3-(4,5-Dimethyl-2-thiazolyl)-2,5-diphenyl-2-H-tetrazolium bromide (MTT) from Invitrogen™ (Thermo Fisher Scientific); dimethylsulfoxide (DMSO) from Dinâmica®; Dulbecco’s modified Eagle’s medium Ham F-12 (DMEM) and Dulbecco’s modified Eagle’s medium high glucose (DMEM HG) from Merk Sigma-Aldrich®; Dulbecco’s phosphate buffered saline (DPBS) from LGC Biotecnologia®; fetal bovine serum (FBS) from Nova Biotecnologia®; and Matrigel® Basement Membrane Matrix from Corning® (Sigma-Aldrich®).

### Melittin

Melittin was provided by the Center for the Study of Venoms and Venomous Animals (CEVAP/UNESP) in purified and lyophilized form. Venom was collected by electrical stimulation, and a reversed-phase binary HPLC system was used for sample profiling and separation. Mass spectrometry analysis was performed on an ESI mass spectrometer (LCQDuo™, ThermoFinnigan, USA). Melittin, after its purification by high-pressure liquid chromatography, was identified by Edman peptide sequencing, and its purity was assessed by mass spectrometry.

### Cell lines and cell culture

The cell lines used were D-17 (ATCC: CCL183), a canine OSA cell line; UMR-106 (ATCC: CRL-1661), a murine OSA cell line; and MG-63 (ATCC: CRL-1427), a human OSA cell line. Cell expansion occurred in 75 cm² culture flasks until they reached 80 to 90% confluence in a controlled humid atmosphere (5% CO2 and 37°C). DMEM Ham's F12 culture medium was used for D-17, and DMEM was used for MG-63 and UMR-106, both added with 10% fetal bovine serum (FBS), 0,5% antibiotic-antimycotic solution, and 1% gentamicin. All the cells tested negative for *Mycoplasma* spp. The cells used were in their logarithmic growth phase in all the experiments.

### *In vitro* cytotoxicity assay

A colorimetric MTT assay was used to determine the cytotoxic effect of melittin. For this purpose, 1x10^4^ D-17, UMR-106, and MG-63 cells/well were seeded in a 96-well plate. The doses tested were 0.5, 1, 2, and 4 μg/mL for UMR-106 and MG-63 and 0.75, 1.5, 3, and 6 μg/mL for D-17. After 24 hours of seeding in DMEM or DMEM HG with 10% FBS, the medium was replaced with medium without FBS plus different concentrations of the tested compounds under the same conditions and for the same period (24 hours). The MTT assay (Invitrogen™, Thermo Fisher Scientific, USA) was performed according to the manufacturer’s instructions, and spectro colorimetric analysis was performed in a microplate reader (570 nm range). All the cell lines were tested at four concentrations via serial dilution, guided by control groups, and executed in triplicate. 

### Cellular proliferation

The potential for the inhibition of cellular proliferation was tested via a wound healing test after treatment with three different doses, 0.5, 1.0, and 1.5 μg/mL. For this purpose, the cells were seeded in a 24-well plate (1.5x10^5^ cells/well) until they reached 90% confluence. After that, a 100 µL pipette tip was used to trace a linear wound. The plates were washed twice with DPBS (500 µL) and agitated for 1 min to remove the attached cells [17].

For reference, each well was photographed at the time the wound was made and after 8, 24 and 32 hours. During this time, the cells were incubated in fresh DMEM or DMEM/HG (without FBS), and the test compound was added at the appropriate dosage. 

### Cellular invasion and migration

The potential for the inhibition of cellular invasion and migration was tested by a Transwell test after treatment with the three different doses, with and without Matrigel®. Uncoated costar transwells (Corning) and Matrigel®-coated transwells (BD Biosciences) were used to detect migration and invasion, respectively. A total of 2x10^4^ cells (4x10^4^ for invasion) in 200 μL of serum-free medium were added to the upper chamber, and various doses of melittin (0.5, 1.0 and 1.5 μg/mL) were added. A total of 600 μL of medium supplemented with 10% FBS, which serves as a chemoattractant, was added to the lower chamber. For the transwell invasion assay, the cultured cells in the plates were pretreated with 50 μL of Matrigel®. The cells were incubated at 37°C and 5% CO2 for 24 h. The cells were fixed with 1 mL of methanol for 10 min. Subsequently, 0.1% crystal violet was added, and the mixture was incubated for 4 min to stain the cells on the lower surface. The cells that did not migrate were cleaned with a cotton swab. The number of cells on the lower surface was counted by imaging under an inverted microscope (magnification, × 20). Three random fields were taken under a microscope and counted. 

### Apoptosis analysis

Cell death analysis of D-17, UMR-106 and MG-63 cells was performed after 24 hours of treatment with 2 μg/mL melittin. A positive control group with only DMEM and a negative control group with 10% DMSO were included. The samples were suspended in the medium containing calcium (buffer solution) for analysis of apoptosis. To this end, 10 µL of APC-conjugated Annexin V (Becton Dickinson and Company) and 10 µL (1.5 µM final concentration) of propidium iodide (Becton Dickinson and Company) were added to the cell suspensions. All samples were incubated in the dark for 10 min at room temperature, and flow cytometry assessment was performed with a final concentration of 1x10^6^ cells/mL in Fortessa LSR equipment (Becton Dickinson, Mountain View, CA, USA). The filter configurations for the PMTs used to measure the fluorescence emission of the applied fluorochromes were 694/50 nm (IP), 660/20 nm (Annexin-APC), and 450/50 nm (Hoechst 33342). The acquisition rate was 800 events per second, and at least 1x10^4^ cells were analyzed per sample. Data were generated in a contour plot graph including axes < 0t (biexponential), making all events visible and properly compensated through BD FACSDiva TM software v6.1 (Becton Dickinson).

### Sarcosphere assay

The cells were seeded in an Ultra-Low Attachment U-shaped plate (Nunclon™, Sphera™ 96well, Nunclon Sphera-Treated, U-Shaped-Botton Microplate, 174925, Thermo Fisher Scientific, USA) with 1x10^4^ D-17, UMR-106, and MG-63 cells/well. After 24 hours of seeding in DMEM (for D17) or DMEM HG (for UMR-106 and MG-63) with 10% FBS, the media was replaced with DMEM without FBS, in three different concentrations (2, 4 and 6 μg/mL) of the tested compounds under the same conditions for the same period (24 hours). The MTT assay (Invitrogen™, Thermo Fisher Scientific, USA) was performed according to the manufacturer’s instructions, and spectro colorimetric analysis was performed in a microplate reader (570 nm range). All the cell lines were tested at three concentrations via serial dilution, guided by control groups, and executed in triplicate. GraphPad Prism 8.0.1 software was used to normalize the Spectro colorimetric data, plot a nonlinear regression, and determine the IC50 via a dose‒response curve. 

### Data analysis

GraphPad Prism 8.0.1 software was used to normalize the spectro colorimetric data, plot a nonlinear regression, and determine the IC_50_ via a dose‒response curve. The values presented are the means and standard deviations of triplicate tests, and statistical significance (p < 0.05) was obtained by a comparison of each tested group with the control (vehicle) group via independent t tests and ANOVA. Wound healing was measured in five different regions via the GIMP 2.10.14 program. The mean distance for each cell line was calculated as the mean and standard deviation. The treated groups and the control were compared via individual t tests when p < 0.05, was obtained by a comparison of each tested group with the control (vehicle) group via independent t tests and ANOVA. The difference in wound healing was calculated via the formula D0-D1, where D0 was the first measurement, and D1 was the final measurement. For the cell migration analysis, the mean and standard deviation were calculated. The treated groups and controls were compared via the Mann‒Whitney test, with p < 0.05 considered an indicator of statistical significance, which was performed with GraphPad Prism 8.0.1 software.

## Results

### *In vitro* cytotoxicity assay

**Figure 1.**
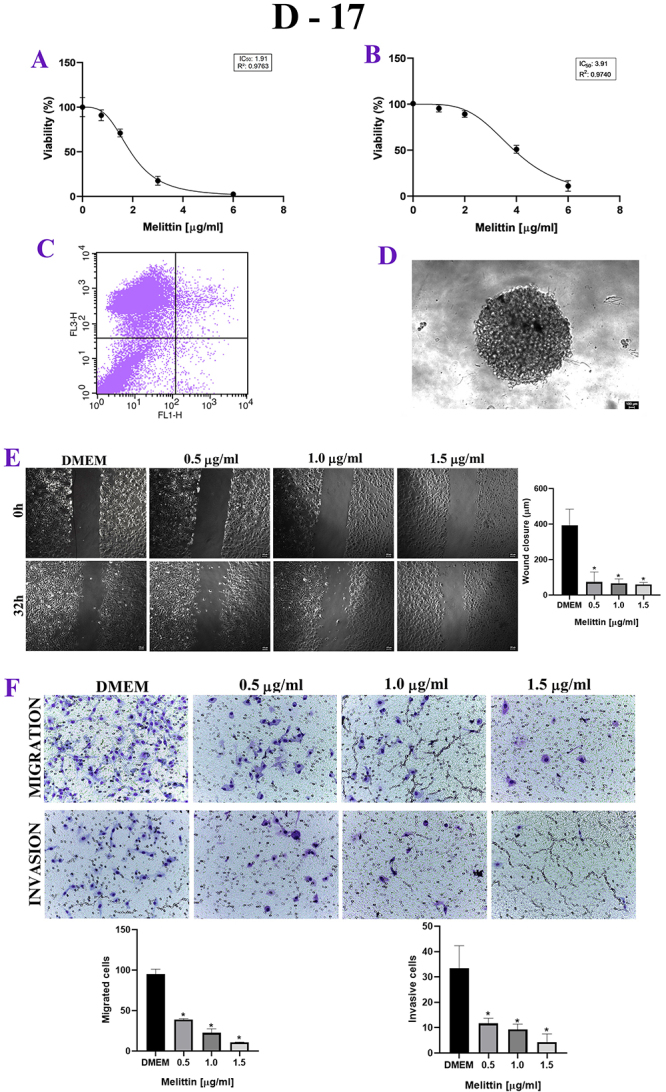
(A) MTT, *in vitro* cytotoxicity assay. X axis: melittin concentration in μg/mL. Y axis: cell viability in percentage. Note the decrease in viability of the human cell line in a dose-dependent manner. (B) Labeling was performed predominantly with propidium iodide, at a dose 2 μg/mL of melittin. (C) Sarcosphere assay (MTT 3D), there is a decrease in viability of canine cell line in a dose-dependent manner. (D) D-17 spheroid with 10 thousand cells. (E) Wound healing assay, in 0h and 32h after the addition of melittin. X axis: melittin concentration in μg/mL. Y axis: wound closure (μm). *p < 0.05. (F) Trasnwell assay. X axis: melittin concentration in μg/mL. Y axis: migrated cells and invasive cells. Note the inhibition of invasion and migration by D-17 under the effect of melittin. *p < 0.05.

**Figure 2.**
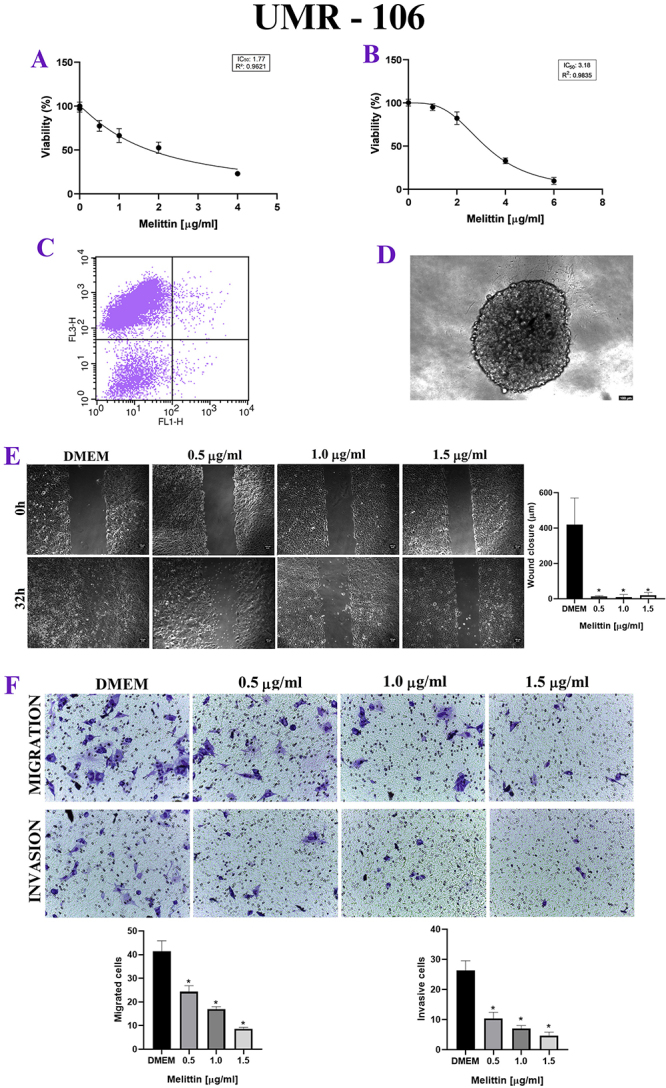
(A) MTT, *in vitro* cytotoxicity assay. X axis: melittin concentration in μg/mL. Y axis: cell viability in percentage. Note the decrease in viability of the human cell line in a dose-dependent manner. (B) Labeling was performed predominantly with propidium iodide, at a dose 2 μg/mL of melittin. (C) Sarcosphere assay (MTT 3D), there is a decrease in viability in murine cell line in a dose-dependent manner. (D) UMR-106 spheroids with 10 thousand cells. (E) Wound healing assay, in 0h and 32h after the addition of melittin. X axis: melittin concentration in μg/mL. Y axis: wound closure (μm). *p < 0.05. (F) Trasnwell assay. X axis: melittin concentration in μg/mL. Y axis: migrated cells and Invasive cells. Note the inhibition of invasion and migration of UMR-106 under the effect of melittin. *p < 0.05.

**Figure 3.**
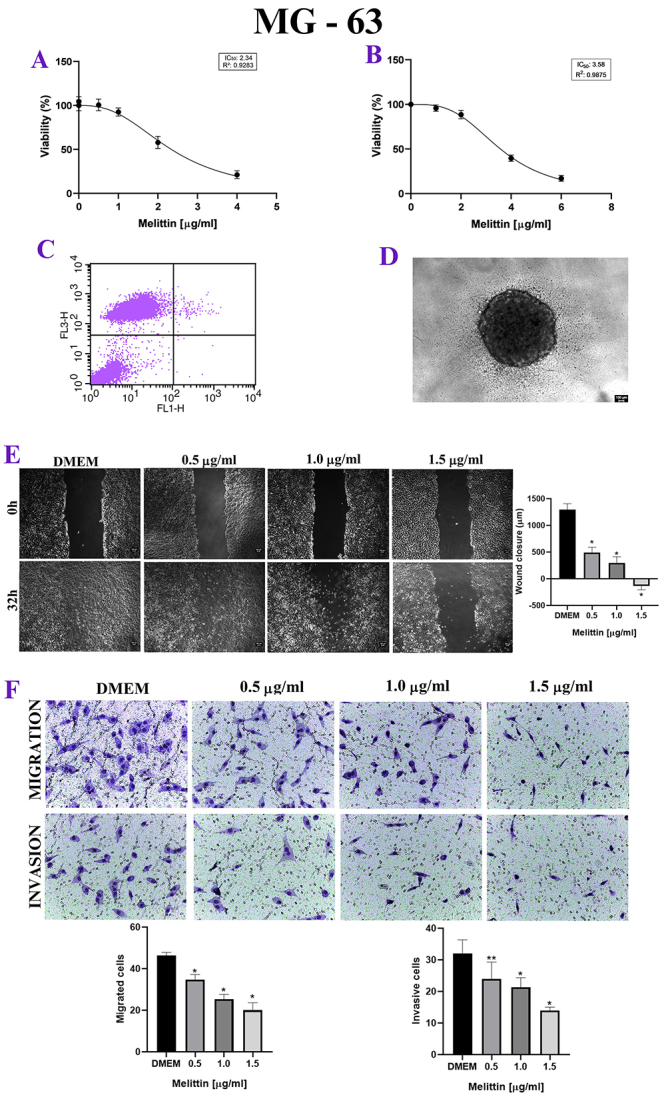
(A) MTT, *in vitro* cytotoxicity assay. X axis: melittin concentration in μg/mL. Y axis: excel viability in percentage. Note the decrease in viability of the human cell line in a dose-dependent manner. (B) Labeling was performed predominantly with propidium iodide, at a dose 2 μg/mL of melittin. (C) Sarcosphere assay (MTT 3D), there is a decrease in viability in human cell line in a dose-dependent manner. (D) MG-63 spheroids with 10 thousand cells. (E) Wound healing assay, in 0h and 32h after the addition of melittin. X axis: melittin concentration in μg/mL. Y axis: wound closure (μm). *p < 0.05. (F) Trasnwell assay. X axis: melittin concentration in μg/mL. Y axis: migrated cells and invasive cells. Note the inhibition of invasion and migration of MG-63 under the effect of melittin, *p < 0.05; **p < 0.05.

For the canine, human and murine osteosarcoma cell lines (D-17, UMR-106 and MG-63), there was a decrease in cellular metabolic activity with increasing melittin concentration in a dose-dependent manner, with reduced cell viability at higher doses of melittin but without much reduction at doses lower than 1 μg/mL. IC_50_ D-17: 1.91 μg/mL. IC_50_ UMR-106: 1.77 μg/mL. There was a significant difference in all groups compared with the control group (DMEM) (p < 0.05); IC_50_ of MG-63: 2.34 μg/mL (Figure 1 A , Figure 2 A, Figure 3 A  and Table 1). 

### Cellular proliferation

For D-17, UMR-106 and MG-63, when the mean degree of wound closure was compared between 0h and 24h, melittin delayed wound healing, with inhibition of proliferation, when compared to the control group, even at lower doses of melittin, such as 0.5 μg/mL (Figure 1 E , Figure 2 E  and Figure 3 E ).

### Cellular invasion and migration

In the Transwell test, melittin inhibited cell invasion and migration in a dose-dependent manner. There was both a decrease in the number of migrated cells and invasive cells when compared to the control group, even at the lowest doses tested, such as 0.5 μg/mL, in canine, murine and human cell lines (Figure 1 F , Figure 2 F  and Figure 3 F ).

### Apoptosis analysis

For the three osteosarcoma cell lines, there was no Annexin labeling in a significant cells percentage, with most cells labeled with propidium iodide, which shows that there was no significant cell death by apoptosis, at a dose 2 μg/mL of melittin. (Figure 1 B , Figure 2 B , Figure 3 B  and Table 2).

**Table 1.** Cell viability, in the MTT test for D-17, UMR-106 and MG-63 cells, in the four different tested doses.

Cell/melittin	1^st^ dose	2^nd^ dose	3^rd^ dose	4^th^ dose
D-17	90.88%	71.10%	17.54%	2.55%
UMR-106	100%	92.52%	57.96%	21.15%
MG-63	77.47%	66.41%	52.61%	21.15%

D-17: canine cell line; UMR-106: murine cell line; MG-63: human cell line.

**Table 2.** Evaluation of the number of cells positive for annexin V (AN) and propidium iodide (PI) in the control, control and cell groups.

Apoptosis Analysis	Control+	D- 17	UMR-106	MG-63	Control-
**PI+/AN−**	2.47%	65.91%	90.97%	87.56%	67.78%
**PI+/AN+**	1.63%	3.03%	0.85%	0.38%	31.40%
**PI−/AN+**	0.39%	0.84%	0.36%	0.03%	0.19%
**PI−/AN−**	95.51%	30.22%	7.82%	12.03%	0.63%

AN: Annexin V; PI: propidium iodide; +: positive; -: negative; PI+/AN−: dead cells; PI+/AN+: late apoptosis; PI−/AN+: early apoptosis; PI−/AN−: viable cells; Control + group: cells with DMEM; Control − group: cells treated with 10% DMSO.

### Sarcosphere assay

In the sarcosphere assay, to test the antitumor potential of melittin in 3D culture, the IC_50_ was greater than that obtained in 2D culture. Has decrease in viability in a dose-dependent manner, but with greater resistance compared with that of monolayer cultivation of the same cell. All groups were compared with the DMEM group, with p < 0.05. IC_50_ D-17: 3.91 μg/mL. IC50 UMR-106: 3.18 μg/mL. IC_50_ of MG-63: 3.58 μg/mL. (Figure 1 C , Figure 2 C  and Figure 3 C).

## Discussion

We used three cell lines to create a complete study model in the context of comparative oncology, as the canine model has proven to be excellent, but many studies with murine models are still being carried out. Melittin presented a similar IC50 among the three species (canine, human and murine) of osteosarcoma cell lines, ranging from 1.5 to 2.5 μg/mL, a relatively low value regarding natural compounds. In the study conducted by Qin *et al.* [18], an IC50 of 6.33 μg/mL was found for UMR-106, and for MG-63, Chu *et al.* [19] reported a dose of 2.8 μg/mL, which is very close to our results. In another study carried out by Zhu *et al.* [20] in another human osteosarcoma cell line (143B), the IC50 was also close to 2.5 µg/mL, which shows that melittin acts similarly among osteosarcoma cells, regardless of the species. We can infer that the mechanism of action of melittin is related to a pathway that is conserved among species.

In the wound healing assay, melittin inhibited cell proliferation, resulting in a reduction in proliferation at doses of 0.5 μg/mL in the cell lines. A similar result was reported by Zhu *et al.* [20] in the human osteosarcoma cell line 143B, where melittin was able to inhibit cell proliferation in the Wound Healing test at doses of 1.0 and 2.0 μg/mL (values below the IC50 found for this cell line), both over a period of 24 and 48 hours. These findings indicate that even at noncytotoxic doses, melittin has potential as an antiproliferative agent.

In the Transwell assay, melittin was able to inhibit both invasion and migration at the lowest doses tested, a similar result to that reported by Qin *et al.* [18] for UMR-106 cells, where melittin was able to inhibit cell migration at doses lower than 50% of the IC50. Zhu *et al.* [20] also reported such effects in 143B cells, with inhibition of migration and invasion at lower doses through a decrease in matrix metalloproteinase-2 (MMP-2) and matrix metalloproteinase-9 (MMP-9). Similar results were also reported in other types of tumors, such as breast tumors [21] and castration-resistant prostate cancer [22], where melittin was able to inhibit cell proliferation and invasion at different doses.

In our study, melittin induced the death of the studied cell lines through propidium iodide marking, indicating that there was no cell death by apoptosis. Similar results were obtained by Mahmoodzadeh *et al.* [23] in AGS cells (human gastric carcinoma), where at doses of 1 and 2 μg/mL, the cells suffered damage to the integrity of the membrane, in addition to not showing an apoptotic pattern of DNA fragmentation, leading to necrotic cell death. Consistent with these results, in the Transwell test, the morphology of the tumor cells disrupted the cell membrane, a feature described in necrotic cells. Apoptotic cells maintain the integrity of the cell membrane and form apoptotic bodies. In contrast, Chu *et al.* [19] and Fan *et al.* [24] demonstrated that MG-63 cells die via apoptosis through the inhibition of the Ca²+ ion pump, which suggests that melittin can induce different cell death mechanisms even in the same cell type. On the other hand, calcium is an important ion that triggers cellular necrosis via the activation of enzymes such as DNase and phospholipase.

In the sarcosphere assay, melittin exhibited a higher IC50 than 2D melittin, which is between 3 and 4 μg/mL. This can be explained by the fact that the cells in the center do not receive oxygen or nutrients to the same extent as the external cells do, leading to a necrotic and hypoxic center, which mimics the tumor microenvironment, which is sometimes hypoxic inside [25], leading to an increase in HIF-α activity, which is correlated with tumor aggressiveness and invasiveness. Some studies have demonstrated that melittin is capable of inhibiting Hypoxia-Inducible Factor-alpha (HIF-α) [26]. Another possible cause is the increased expression of P-glycoprotein, a protein that is related to resistance to multiple drugs and has already been found to be increased in several cell types in 3D models compared with 2D models, leading to a decrease in drug accumulation in cells [25, 27]. Another factor may be the difference in melittin, which reaches cells on the periphery and cells in the center, causing greater resistance in the arrival of the compound to the center of the spheroid, which may explain the greater plateau observed in the 3D viability graphs than in the 2D viability graphs.

This disparity between toxicity in 2D and 3D models has already been reported by several authors [25, 27, 28]. In cell cultures such as breast tumors, lung tumors, multiple myeloma and compounds such as gemcitabine, 5-FU, doxorubicin, metrotezxane, bortezomib and carfilzomib indicate that cells in the 3D model have greater innate resistance to anticancer agents than those in the 2D model do [27].

3D study models are extremely important in oncology, as they mimic the tumor microenvironment in terms of physical and biological characteristics, in addition to their similar topology growth, gene expression, and metabolism, surpassing 2D models [28]. This is the first study carried out with melittin in 3D culture, which could be interesting in research into the use of melittin directed toward tumor target cells without damaging other cell types, given that melittin presents certain toxicity to peripheral blood cells [29]. 

The use of nanocarriers [25, 30, 31], could be an alternative for direct delivery of the drug to the tumor site without exerting its cytotoxic potential. A study with nanocarriers in murine tumors *in vivo* revealed a significant reduction in tumor growth without signs of toxicity, indicating that nanocarriers are capable of selectively delivering melittin to tumor targets [25]. Melittin has the potential to inhibit proliferation, invasion and migration even at low doses, making this peptide very promising for its use as an antitumor drug associated with the use of nanocarriers, which deliver melittin directly to the tumor site. 

## Conclusion

We confirmed that melittin exerts a cytotoxic effect on canine, murine and human osteosarcoma cell lines, in addition to inhibiting cell proliferation, migration and invasion in 2D culture. Even at the lowest doses, it also exerts a cytotoxic effect in 3D culture, which highlights the great potential of this compound as a possible antitumor drug. Further research is necessary to explore its mechanisms of action so that it can be used in the development of drugs for the treatment of cancers that are unresponsive to current treatments.

### Abbreviations

DMEM HG: Ddulbecco’s modified Eagle’s medium high glucose; DMEM: Dulbecco’s modified Eagle’s medium Ham's F12; DMSO: dimethylsulfoxide; DPBS: dulbecco’s phosphate-buffered saline; FBS: fetal bovine sérum; OSA: osteosarcoma.

## Data Availability

The datasets generated during and/or analyzed during the current study are available from the corresponding author upon reasonable request.
